# Combined detection of serum autoantibodies as diagnostic biomarkers in esophagogastric junction adenocarcinoma

**DOI:** 10.1007/s10120-018-0894-y

**Published:** 2018-11-13

**Authors:** Yi-Wei Xu, Hao Chen, Hai-Peng Guo, Shi-Han Yang, Yu-Hao Luo, Can-Tong Liu, Xin-Yi Huang, Xue-Miao Tang, Chao-Qun Hong, En-Min Li, Li-Yan Xu, Yu-Hui Peng

**Affiliations:** 1 Department of Clinical Laboratory Medicine, The Cancer Hospital of Shantou University Medical College, Shantou, 515041 People’s Republic of China; 2 Guangdong Esophageal Cancer Research Institute, Shantou University Medical College, Shantou, 515041 People’s Republic of China; 3 The Key Laboratory of Molecular Biology for High Cancer Incidence Coastal Chaoshan Area, Shantou University Medical College, Shantou, 515041 People’s Republic of China; 4 State Key Laboratory of Oncology in South China, Collaborative Innovation Center for Cancer Medicine, Sun Yat-Sen University Cancer Center, Guangzhou, 510060 People’s Republic of China; 5 Department of Head and Neck Surgery, The Cancer Hospital of Shantou University Medical College, Shantou, 515041 People’s Republic of China; 6 Department of Dermatology and Venereology, Shantou Central Hospital, Shantou, 515041 People’s Republic of China; 7 Department of Oncological Laboratory Research, The Cancer Hospital of Shantou University Medical College, Shantou, 515041 People’s Republic of China; 8 Department of Biochemistry and Molecular Biology, Shantou University Medical College, Shantou, 515041 People’s Republic of China; 9 Institute of Oncologic Pathology, Shantou University Medical College, Shantou, 515041 People’s Republic of China

**Keywords:** Autoantibody, Tumor-associated antigen, Diagnosis, Esophagogastric junction adenocarcinoma

## Abstract

**Background:**

We previously found that autoantibodies against a panel of six tumor-associated antigens (p53, NY-ESO-1, MMP-7, Hsp70, PRDX6 and Bmi-1) may aid in early detection of esophageal squamous cell carcinoma. Here we aimed to evaluate the diagnostic value of this autoantibody panel in esophagogastric junction adenocarcinoma (EJA) patients.

**Methods:**

Serum autoantibody levels were measured by enzyme-linked immunosorbent assay in a training cohort and a validation cohort. We used receiver-operating characteristics (ROC) to calculate diagnostic accuracy.

**Results:**

We recruited 169 normal controls and 122 EJA patients to the training cohort, and 80 normal controls and 70 EJA patients to the validation cohort. Detection of the autoantibody panel demonstrated an area under the curve (AUC) of 0.818, sensitivity 59.0% and specificity 90.5% in training cohort, and AUC 0.815, sensitivity 61.4% and specificity 90.0% in validation cohort in the diagnosis of EJA. Measurement of the autoantibody panel could distinguish early stage EJA patients from normal controls (AUC 0.786 and 0.786, sensitivity 50.0% and 56.0%, and specificity 90.5% and 90.0%, for training and validation cohorts, respectively). Moreover, a restricted panel consisting of autoantibodies against p53, NY-ESO-1 and Bmi-1 exhibited similar diagnostic performance for EJA (AUC 0.814 and 0.823, sensitivity 53.5% and 60.0%, and specificity 90.5% and 93.7%, for training and validation cohorts, respectively) and early stage EJA (AUC 0.744 and 0.773, sensitivity 55.6% and 52.0%, and specificity 90.5% and 93.7%, for training and validation cohorts, respectively).

**Conclusions:**

Autoantibodies against an optimized TAA panel as serum biomarkers appear to help identify the present of early stage EJA.

**Electronic supplementary material:**

The online version of this article (10.1007/s10120-018-0894-y) contains supplementary material, which is available to authorized users.

## Introduction

In recent years there has been an alarmingly rising incidence of esophagogastric junction adenocarcinoma (EJA) in both Western countries and Eastern Asian [1–3]. EJA derives from epithelial tissue of esophagogastric junction and crosses the esophagogastric junction line, regardless of the location of the tumor epicenter is at the distal esophagus or proximal stomach. EJA has been regarded as a separate entity with distinct features in the aspects of genetics, epidemiology and prognosis. Despite attempts to improve, the classification, diagnosis and treatment strategy for EJA remain controversial. The majority of EJA patients are often diagnosed at an advanced stage thus with a fatal prognosis, due to the absence of typical symptoms at the early stage of oncogenesis [4]. In addition, EJA is found to have an early risk of extensive metastases to the mediastinal and abdominal lymph nodes, which also lead to poor outcomes of patients [5–7]. Thus, the exploration of effective and reliable methods to identify EJA at an early stage is the key to improving the survival of patients with this disease.

Autoantibodies against tumor-associated antigens (TAAs) were initially identified in the sera of melanoma patients in 1977 [8], and have drawn significant attention as they have created chances to develop a source of biomarkers based on the immune system and could be detected at early onset of the cancer disease. Over the past two decades, many studies have demonstrated the potential utility of autoantibodies for cancer lies in the role of early detection, which might supplement current screening strategies to aid early cancer diagnosis [9–11]. However, the relationship between sera autoantibodies and EJA has not been well characterized. Only a study reported by Zhou et al. evaluated serum autoantibodies to a panel of seven tumor-associated antigens (C-myc, IMP1, Koc, p16, p53, p62 and Survivn) in the patients with EJA and indicated that autoantibodies might be useful to differentiate patients with EJA from normal controls [12].

We have recently reported that a panel autoantibody against six TAAs (p53, NY-ESO-1, MMP-7, Hsp70, PRDX 6 and Bmi-1) might be used as a blood biomarker-based tool to identify early stage esophageal squamous cell carcinoma (ESCC) and Nasopharyngeal Carcinoma [13, 14]. In this study, we applied these autoantibody biomarkers to EJA patients collected from two centers (i.e., a training cohort and a validation cohort) and assessed whether these autoantibodies have diagnostic value for EJA.

## Materials and methods

### Study samples

We performed a retrospective study to evaluate the diagnostic value of autoantibodies for EJA. The EJA patients and healthy volunteers from the Cancer Hospital, Shantou University Medical College, from September 2012 to June 2017 were recruited as training cohort. A validation cohort comprising sera of EJA patients and healthy volunteers were collected from the Sun Yat-Sen University Cancer Center, from January 2017 to May 2018. Eligible patients had gastroscopy, spiral computed tomography and histopathological examination as EJA without previously suffering from any cancer disease, and did not receive any anti-cancer treatment. Healthy volunteers as normal controls, who had medical check-up without evidence of any neoplasm, were obtained from Physical Examination Center in the same hospital. Tumor stage was evaluated according to 8th edition of the American Joint Committee on Cancer (AJCC) Cancer Staging Manual. In the present study, tumors with AJCC stages I + II were defined as early stage EJA.

Peripheral blood samples of EJA patients and controls were allowed to clot at room temperature for 30 min and centrifuged at 1250*g* for 5 min. Then the serum was removed and stored at − 80 °C in the biobank. Informed consent of all participants in this study was obtained prior to the use of the serum samples. This study was complied with principles of the Helsinki Declaration and was approved by the institutional ethics review committee at each center.

### Recombinant proteins expression

The coding sequence regions for P53 (NM_001276760.1), NY-ESO-1 (NM_001327.2), PRDX6 (NM_004905.2), BMI1 (NM_005180.8), MMP7 (NM_002423.3), and HSP70 (NM_005345.5) were subcloned into the pDEST17 expression vector (Invitrogen, Waltham, MA). We conducted the expression, purification, and analysis of these recombinant proteins as described in our previous studies [13, 14].

### Enzyme-linked immunosorbent assay (ELISA) for autoantibody detection

ELISA was performed by two researchers (Yi-Wei Xu and Yu-Hui Peng) that were blind to clinical information as previously described [13, 14]. Briefly, purified recombinant antigens of p53, NY-ESO-1, MMP-7, Hsp70, PRDX6, and Bmi-1 were diluted in 50 mM bicarbonate buffer (pH 9.6) to 0.1, 0.1, 0.6, 0.8, 1.5, and 0.6 mg/mL, respectively. Serum samples and quality control samples (QCS, a pooled serum sample collected randomly from 100 patients with ESCC) were diluted 1/110 in blocking buffer, then were incubated at 37 °C for 1 h, as well as were appropriate control rabbit polyclonal antibodies (Immunosoft, Zhoushan, China) specific for capture proteins. After washing, horseradish peroxidase (HRP)-conjugated goat anti-human IgG or anti-rabbit IgG (Santa Cruz Biotechnology, Santa Cruz, CA) were used as secondary antibodies. After incubation, the plates were washed, and ready prepared 3,3′,5,5′-tetramethylbenzidine (TMB, InTec PRODUCTS, Xiamen, China) and hydrogen peroxide (InTec PRODUCTS) were added. After color formation, the absorbance of each well was read at 450 nm and referenced to 630 nm by a plate microplate reader (Thermo Fisher Scientific, Boston, USA).

All cancer and normal samples were run in duplicate. QCSs were run to ensure quality control monitoring of the assay runs using Levey–Jennings plots. With the purpose of minimizing an intra-assay deviation, the ratio of the difference between duplicated sample OD values to their sum was used to assess precision of the assay. If the ratio was > 10%, the sample test was deemed to be invalid and this sample was retested.

### Immunohistochemistry analysis for TAA

Immunohistochemistry was performed using 2-step protocol according to the manufacturer’s instructions (PV-9000 Polymer Detection System, ZSGB-BIO, Beijing, China) as described previously [15]. Rabbit polyclonal antibodies against p53, NY-ESO-1, MMP-7, Hsp70, PRDX6, and Bmi-1 (all 1:200; Immunosoft, Zhoushan, China) were incubated overnight at 4 °C. A staining index (values 0–12) was calculated by multiplying the two following scores. One score was given according to the intensity of staining: 0, no staining; 1, weak staining; 2, moderate staining; and 3, strong staining; and another score was the percent of positive cells: 1, 0–25% of the cells; 2, 25–50% of the cells; 3, 51–75% of the cells; 4, 75–100% of the cells. Scores of 0–4 were considered weak staining, scores of 5–8 were considered moderate staining, and scores of 9–12 were considered intense staining. When the final score was equal or more than 5, it was considered high expression; otherwise, it was considered low expression.

### Statistical analysis

All analyses were done with SPSS or GraphPad Prism software. We used the Mann–Whitney’s *U* test for analyses that compared different markers between two groups. Receiver-operating characteristic (ROC) analysis was performed to assess the diagnostic parameters including the area under the ROC curve (AUC) with 95% confidence interval (CI), the sensitivity and the specificity. The cut-off value for positive reactivity was evaluated by achieving the maximum sensitivity when the specificity was > 90%, and by minimizing the distance of the cut-off value to the top-left corner of the ROC curve. We selected a specificity of > 90% to produce a test that could be beneficial to early cancer detection [16]. We used a logistic regression model to estimate functions of the combined autoantibody biomarkers or identify optimized autoantibody biomarkers based on the dataset from all the EJA patients and normal controls. The predicted probability of being diagnosed with EJA was treated as a surrogate marker to construct ROC curve [17]. For the improvement of clinical diagnosis interpretation, the positive predictive value (PPV), negative predictive value (NPV), positive likelihood ratio (PLR), and negative likelihood ratio (NLR) were also presented. Wilcoxon signed-ranks test was performed to compare pre-operative and post-operative levels of autoantibodies in EJA patients. Cumulative patient survival time was estimated by the Kaplan–Meier method and compared by the log-rank test. Overall survival (OS) was defined as the interval between the date of tumor resection and death. The data were censored for patients who survived at the last follow-up. In all statistical tests, *p* values were two sided and were considered significant if lower than 0.05.

## Results

### Individual autoantibody levels in EJA

**Fig. 1:**
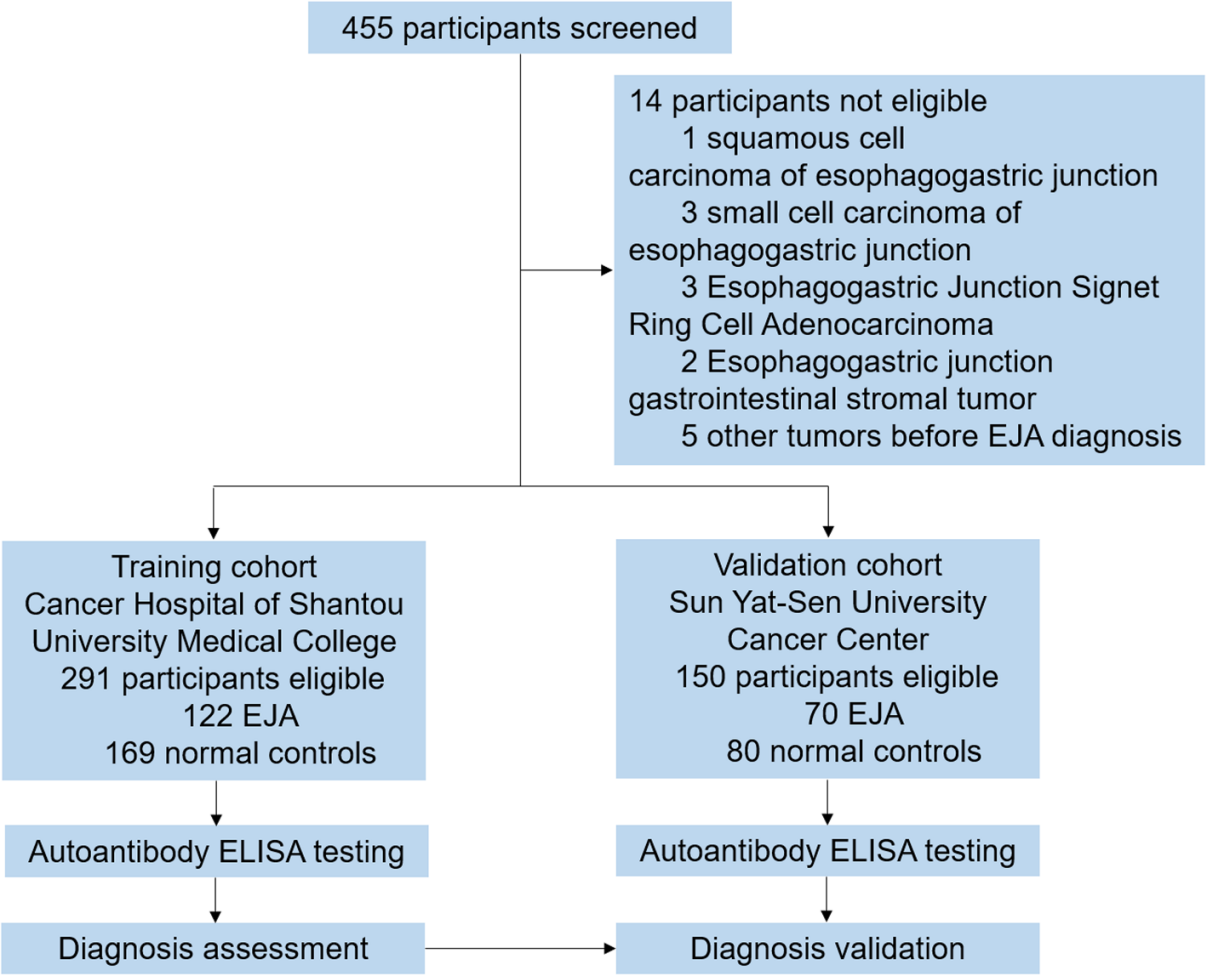
Study profile. EJA, esophagogastric junction adenocarcinoma

In total, there were 441 participants selected in this study, including 122 EJA cases and 169 healthy volunteers in the training cohort and 70 EJA cases and 80 healthy volunteers in the validation cohort (Fig. 1). Clinical features on patient and normal control are shown in Table 1.

**Table 1:** Characteristics of the study population

Group	Training cohort				Validation cohort			
	EJA (*n* = 122)		Normal (*n* = 169)		EJA (*n* = 70)		Normal (*n* = 80)	
	NO.	%	NO.	%	NO.	%	NO.	%
Age in years								
Mean ± SD	64 ± 8		62 ± 9		65 ± 8		61 ± 7	
Range	38–82		40–79		47–85		45–80	
Gender								
Male	103	89.3	141	83.4	55	78.6	58	72.5
Female	19	10.7	28	16.6	15	21.4	22	27.5
TNM stage								
I	2	1.6			11	15.7		
II	16	13.1			14	20.0		
III	87	71.3			30	42.9		
IV	17	13.9			15	21.4		
Histological grade								
High (Grade 1)	14	11.5			7	10.0		
Middle (Grade 2)	44	36.1			26	37.1		
Low (Grade 3)	47	38.5			24	34.3		
Unknown	17	13.9			13	18.6		
Depth of tumor invasion								
T1	2	1.6			9	12.9		
T2	3	2.5			8	11.4		
T3	29	23.8			38	54.3		
T4	88	72.1			15	21.4		
Lymph node metastasis								
Positive	92	75.4			45	64.3		
Negative	30	24.6			25	35.7		
Size of tumor								
≤ 5 cm	49	40.2			37	52.9		
> 5 cm	65	53.3			23	32.9		
Unknown	8	6.5			10	12.3		

**Fig. 2:**
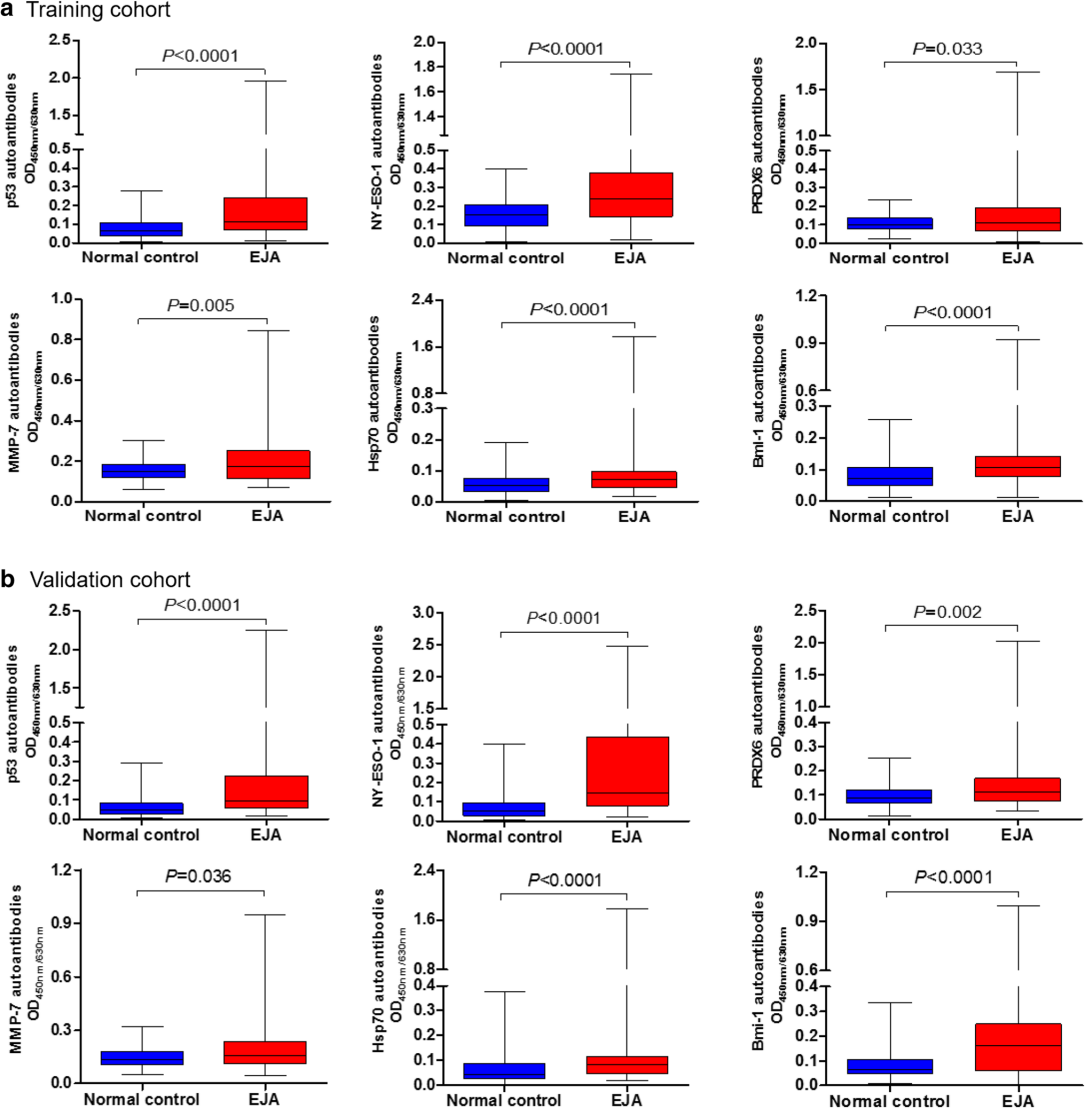
Serum autoantibody levels. Median levels and interquartile ranges of individual autoantibodies in sera of EJA patients and normal controls in the training cohort (**a**) and the validation cohort (**b**) are illustrated by box plot and the whiskers show minimum and maximum value. Mann–Whitney *U* test was conducted to assess differences of autoantibody levels between sera of EJA patients and normal controls. *EJA* esophagogastric junction adenocarcinoma

We tested the presence of autoantibodies against p53, NY-ESO-1, PRDX 6, MMP-7, Hsp70, and Bmi-1 in sera of EJA patients and healthy volunteers by ELISA, and the results demonstrated that in both cohorts serum levels of the six autoantibodies were all elevated in patients with EJA compared to the control group (Fig. 2).

According to ROC analysis, the diagnostic performances of autoantibodies against p53, NY-ESO-1, PRDX 6, MMP-7, Hsp70, and Bmi-1, measured by AUC, were 0.718, 0.718, 0.573, 0.597, 0.652 and 0.686 in the training cohort, respectively, with the corresponding cut-off values of 0.147, 0.279, 0.158, 0.234, 0.117 and 0.147, respectively (Supplementary Fig. 1). The sensitivities against a specificity of > 90% in individual biomarkers in the training cohort ranged from 18.0% (Hsp70 autoantibody) to 37.7% (NY-ESO-1 autoantibody; Supplementary Table 1). We further investigated six autoantibodies in early stage EJA patients, and we found similar AUC values, sensitivities and specificities in patients at such stage to those in all patients with EJA (Supplementary Table 1). Moreover, the diagnostic results of individual autoantibodies for all EJA patients and early stage patients were validated in the validation cohort (Supplementary Fig. 1, Supplementary Table 2).

### Autoantibody panel in EJA

**Fig. 3:**
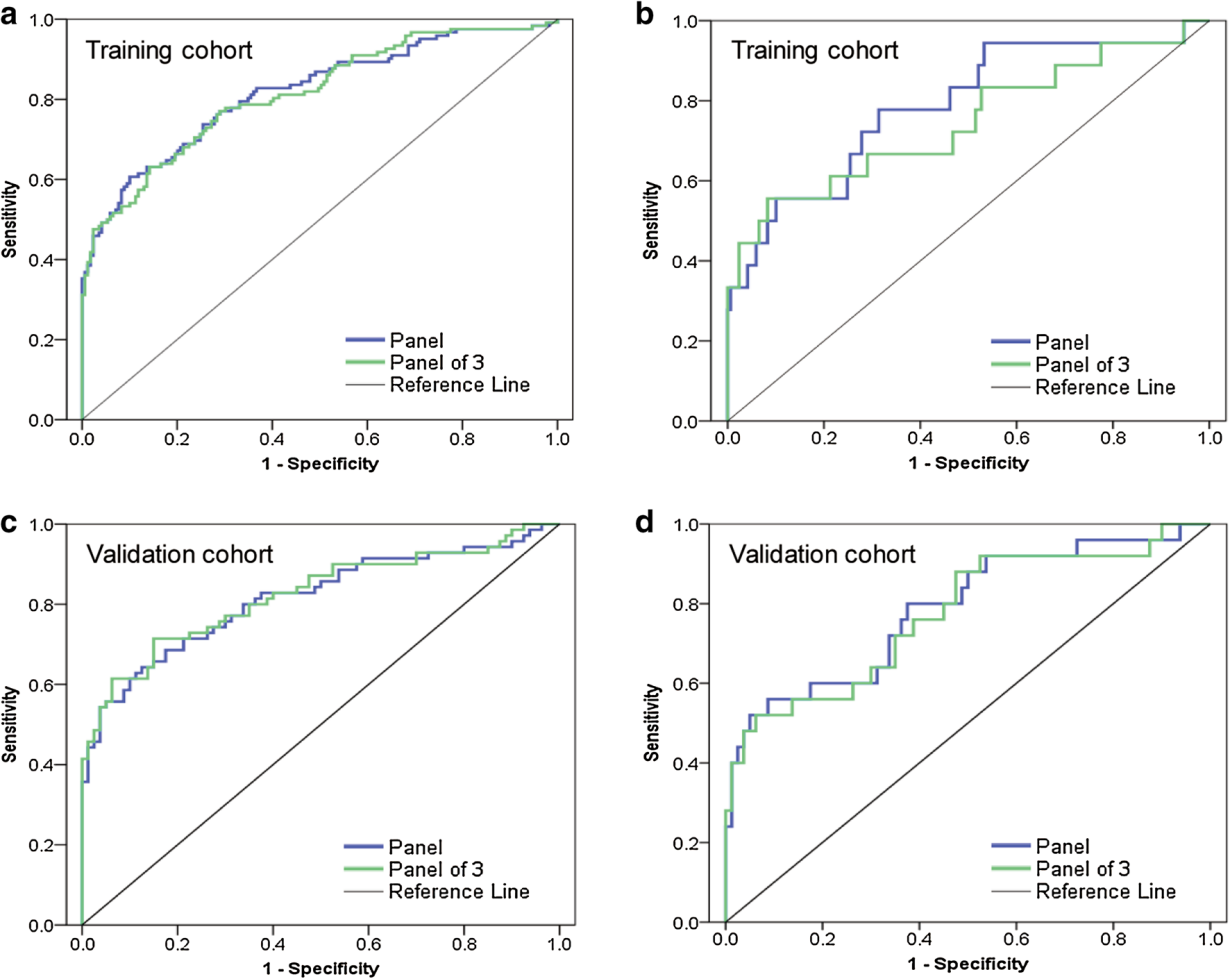
Performance of the autoantibody panel and panel of 3 to detect EJA. **a** ROC curve for the autoantibody panel and panel of 3 for all patients with EJA vs. normal controls in training cohort. **b** ROC curve for the autoantibody panel and panel of 3 for patients with early stage EJA vs. normal controls in training cohort. **c** ROC curve for the autoantibody panel and panel of 3 for all patients with EJA vs. normal controls in validation cohort. **d** ROC curve for the autoantibody panel and panel of 3 for patients with early stage EJA vs. normal controls in validation cohort. Panel: autoantibodies against six tumor-associated antigens. Panel of 3: autoantibodies against p53, NY-ESO-1 and Bmi-1. EJA, esophagogastric junction adenocarcinoma

A binary logistic regression analysis (method = ENTER) to score the risk of being diagnosed with EJA was applied on the dataset comprised of 291 serum samples from EJA patients and normal controls in the training cohort. The predicted probability (*p*) for EJA from the logit model on the basis of the autoantibody panel against six antigens was calculated by ln[*p*/(1 − *p*)] = 7.033 × (p53) + 4.731 × (NY-ESO-1) + 1.761 × (PRDX6) + 2.516 × (MMP7) + 3.764 × (Hsp70) + 4.910 × (Bmi-1) − 3.557, and was used to establish the ROC curve (Fig. 3). With the use of ROC analysis and a cut-off *p* value of 0.505, the AUCs for the autoantibody panel were 0.818 (95% CI, 0.767 to 0.869) in the training cohort and 0.815 (95% CI, 0.744–0.866) in the validation cohort (sensitivity 59.0% and specificity 90.5% in the training cohort; sensitivity 61.4% and specificity 90.0% in the validation cohort; Table 2). The performance of the autoantibody panel in distinguishing the group of early stage EJA patients from the normal control group was further evaluated (Fig. 3). The analysis showed that the autoantibody panel also had diagnostic value in differentiating early stage EJA from normal controls (AUC 0.786, 95% CI 0.665–0.908, sensitivity 50.0%, specificity 90.5% in the training cohort; AUC 0.786, 95% CI 0.677–0.896, sensitivity 56.0%, specificity 90.0% in the validation cohort; Table 2).

**Table 2:** Diagnostic results for the autoantibody panel and panel of 3 in EJA

	AUC (95% CI)	Sensitivity (%)	Specificity (%)	PPV (%)	NPV (%)	PLR	NLR
Training cohort							
EJA vs. NC							
Panel	0.818 (0.767–0.869)	59.0	90.5	81.7	75.4	6.21	0.45
Panel of 3	0.814 (0.763–0.864)	53.5	90.5	80.2	73.0	5.63	0.51
Early stage EJA vs. NC							
Panel	0.786 (0.665–0.908)	50.0	90.5	35.8	94.5	5.26	0.55
Panel of 3	0.744 (0.600–0.888)	55.6	90.5	38.3	95.0	5.85	0.49
Validation cohort							
EJA vs. NC							
Panel	0.815 (0.744–0.866)	61.4	90.0	84.3	72.7	6.14	0.43
Panel of 3	0.823 (0.753–0.892)	60.0	93.7	89.3	72.8	9.52	0.43
Early stage EJA vs. NC							
Panel	0.786 (0.677–0.896)	56.0	90.0	63.6	86.8	5.60	0.49
Panel of 3	0.773 (0.660–0.887)	52.0	93.7	72.1	86.2	8.25	0.51

Panel: autoantibodies against six tumor-associated antigens

Panel of 3: autoantibodies against p53, NY-ESO-1 and Bmi-1

*CI* exact confidence interval; *EJA* esophagogastric junction adenocarcinoma; *NC* normal controls; *NLR* negative likelihood ratio; *NPV* negative predictive value; *PLR* positive likelihood ratio; *PPV* positive predictive value

To explore whether all the six autoantibodies of the panel required for its diagnostic value, we applied a forward stepwise logistic regression analysis in the training cohort to assess the risk of being diagnosed with EJA. The result showed that autoantibodies against p53, NY-ESO-1 and Bmi-1 turned out to be significant predictors, with the predicted probability of being detected as EJA calculated by ln[*p*/(1 − *p*)] = 8.040 × (p53) + 4.905 × (NY-ESO-1) + 7.946 × (Bmi-1) − 3.081. Similarly, we used the predicted probability to construct the ROC curve (Fig. 3). The optimized autoantibody panel (i.e., panel of 3), when the cutoff was defined as 0.498, had an AUC of 0.814 (95% CI 0.763–0.864) to discriminate patients with EJA from normal controls with a slightly reduced sensitivity of 53.5% and a specificity of 90.5% in the training cohort (Table 2). In the validation cohort, AUC of the panel of 3 was 0.823 (95% CI 0.753–0.892) with 60.0% sensitivity and 93.7% specificity (Fig. 3; Table 2). Similar data were observed in panel of 3 when comparing early stage EJA patients with the normal control group in both cohorts (Fig. 3; Table 2).

We next analyzed the correlation of the autoantibody panel and panel of 3 with clinicopathological features in EJA patients. We found that in the training or validation cohorts the autoantibody panel or panel of 3 did not significantly correlate with age, gender, tumor size, histological grade, depth of tumor invasion, lymph node status or TNM (Supplementary Tables 3 and 4).

### Post-operative autoantibody levels in EJA patients

**Fig. 4:**
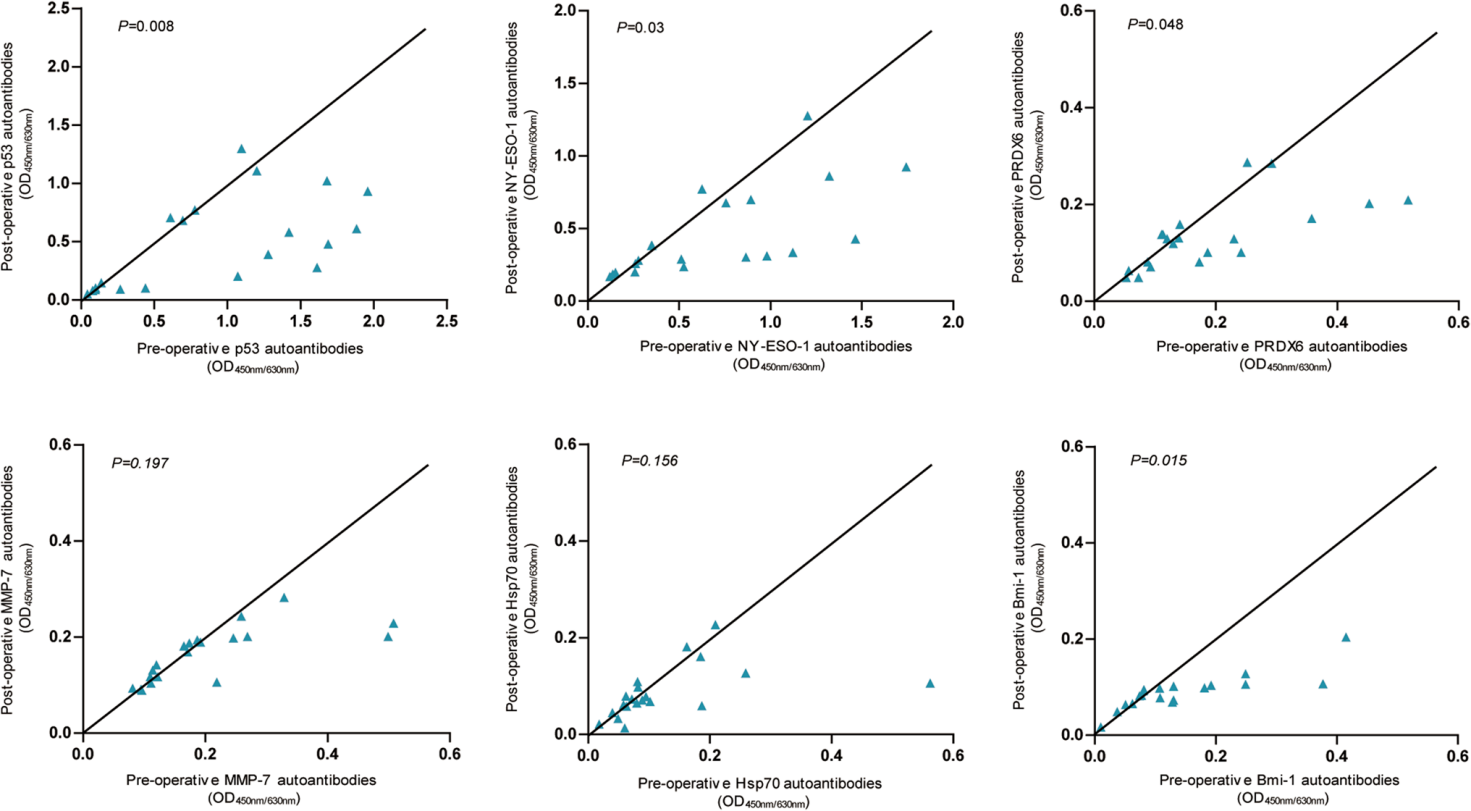
Scatter plot of individual autoantibodies in paired serum samples at pre-operation and 4–6 weeks post-operation from the same patients with EJA. Wilcoxon signed-ranks test was performed to compare pre-operative and post-operative levels of autoantibodies in EJA patients. *EJA* esophagogastric junction adenocarcinoma

We collected paired pre-operative and post-operative serum samples from 20 EJA patients with pre-operative autoantibody panel positivity to assess the changes in individual autoantibodies or the autoantibody panel. The levels of autoantibodies against p53, NY-ESO-1, PRDX6 and Bmi-1 at 4–6 weeks post-operation were drastically lower, respectively, compared with the corresponding pre-operate levels (Fig. 4). In contrast, there was no significant difference between pre-operative and post-operative serum levels of autoantibodies against MMP-7 or Hsp70 (Fig. 4). In addition, the autoantibody panel and panel of 3 after tumor resection became negative in six and five patients, respectively, among these 20 patients (*p* = 0.020 and *p* = 0.047, respectively).

### Survival analysis of the autoantibody panel in EJA

In the training cohort, there were 78 patients resected without neoadjuvant treatment and with complete follow-up data. The maximum and the mean follow-up time for EJA patients’ overall survival were 66.8 months and 45.8 months, respectively, and 34 cases of patients (43.6%) died during the follow-up period. Kaplan–Meier analysis and log-rank test did not show statistically significant differences of 5-year overall survival rates between patients with positive autoantibody panel and negative autoantibody panel (55.0% vs. 54.9%, *p* > 0.05, Supplementary Fig. 2). Similar results were observed in survival analysis of the autoantibody panel of 3 and individual autoantibodies in these same patients with EJA (all *p* > 0.05, Supplementary Fig. 2).

### Expression of individual TAAs in EJA tissue

**Fig. 5:**
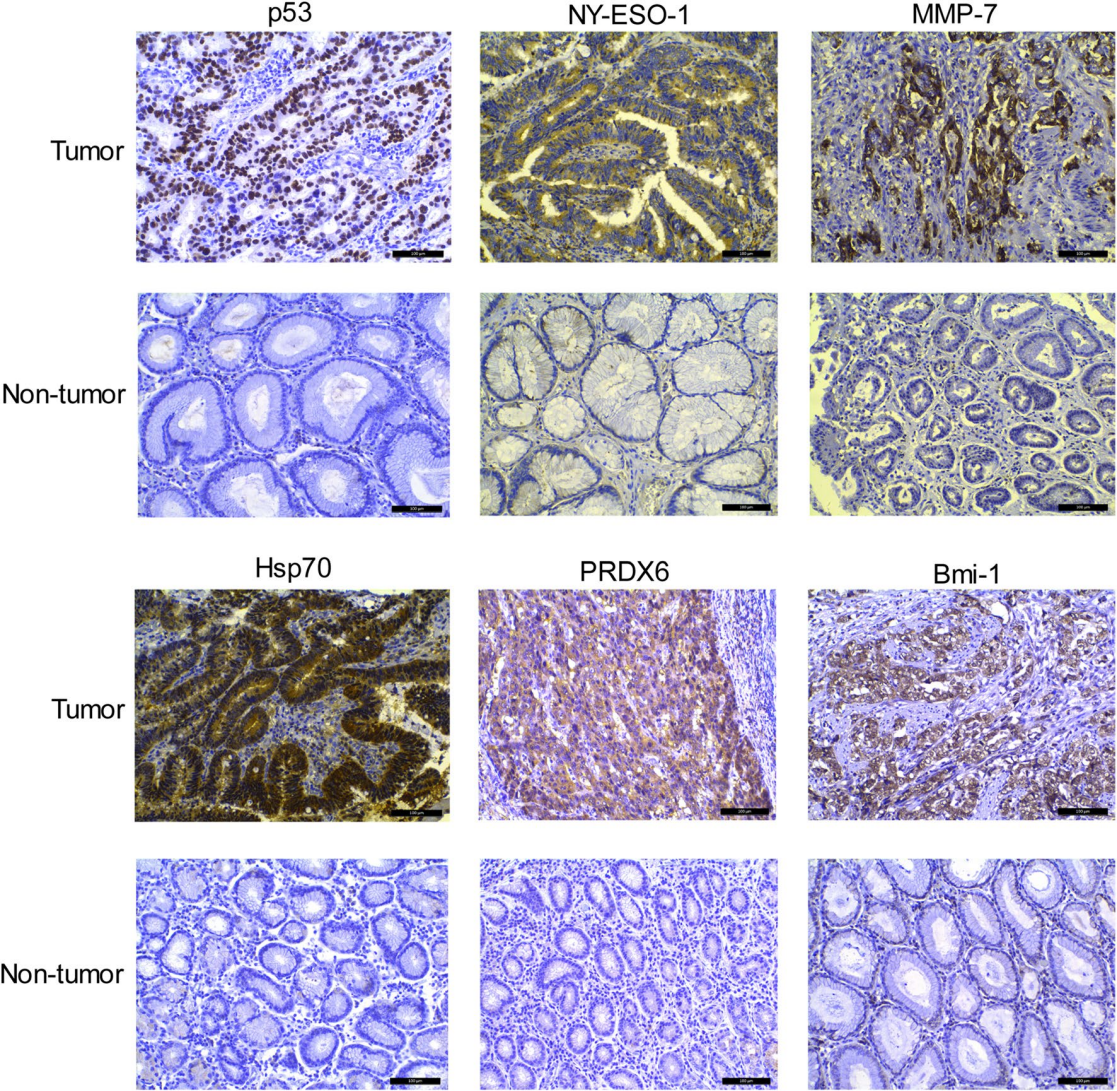
Expressions of p53, NY-ESO-1, MMP-7, Hsp70, PRDX6, and Bmi-1 by immunohistochemistry in representative EJA and paired adjacent non-tumor tissue samples (200 × magnification). Scale bar = 100 µm; *EJA* esophagogastric junction adenocarcinoma

The expressions of p53, NY-ESO-1, MMP-7, Hsp70, PRDX6, and Bmi-1 in tumor cell were detected by immunohistochemistry in 10 tissue samples with EJA and paired adjacent non-tumor tissue samples. As shown in Fig. 5, individual TAAs exhibited similar trend that the expression level was higher in EJA tissues than that in non-tumor tissue samples. Higher expression of p53, NY-ESO-1, MMP-7, Hsp70, PRDX6, and Bmi-1 proteins was observed in 7, 8, 7, 8, 6 and 8 of the 10 tumor tissue samples, respectively, compared to individual TAAs (0, 0, 1, 2, 1 and 1, respectively) in corresponding adjacent non-tumor tissues. These results indicated that the antigenicity to the above individual TAAs may originate from its aberrant expression. In addition, various patterns of TAAs expression were observed in EJA. The positive immunostaining of p53 was constitutively observed in the cell nucleus, and NY-ESO-1, MMP-7 and PRDX6 were located in the cytoplasm; whereas Hsp70 and Bmi-1 showed positive immunostaining in both cell nucleus and cytoplasm.

## Discussion

In this study, we examined the levels of autoantibodies against six antigens p53, NY-ESO-1, PRDX 6, MMP-7, Hsp70, and Bmi-1 in sera from EJA patients and normal controls, and the analysis revealed that all these autoantibodies were potential circulating diagnostic biomarkers for EJA. The autoantibody panel with the six autoantibodies or the optimized autoantibody panel against the three antigens (p53, NY-ESO-1 and Bmi-1) demonstrated acceptable accuracy in the diagnosis of EJA, especially for early stage patients. The diagnostic values of this autoantibody panel and panel of 3 were verified in the training cohort of 122 patients and 169 controls and in the independent validation cohort of 70 patients and 80 controls.

Early detection is supposed to be one of the most promising methods to reduce cancer mortality and cancer burden [18]. In clinical practice, current tool for the early diagnosis of EJA falls into endoscopy [19]. However, the invasive nature of this modality makes it hard to be acceptable, particularly for the screening of the asymptomatic population. On the other hand, it may be difficult to diagnose early lesions of EJA, as the endoscopist may not have the ability to identify precancerous lesions such as dysplastic areas of columnar mucosa or areas of mucosal thickening, or early cancer. It has been proposed that the best hope for earlier cancer detection lies in biomarker. The identification and development of robust biomarkers that could be detected in blood or urine samples might assist in the risk prediction and early detection of cancer [20, 21]. In recent years, many discoveries of serum tumor biomarkers identified by genomic and proteomic techniques have been documented, such as miRNAs [22], Long noncoding RNAs [23], Circulating tumor DNAs [24], circulating tumor cells [25], and metabolites [26]. However, few of these biomarkers for early cancer detection have surpassed blinded Phase III validation studies and have been applied to the clinic over the past two decades [11, 27, 28]. Accumulating evidence of circulating serum autoantibodies in cancer patients highlighted the potential use of autoantibody in early detection [29, 30]. Serum autoantibodies could appear even before the development of clinical symptoms [10, 31–33]. Importantly, EarlyCDT®-Lung Test, a panel of autoantibody-based diagnostic tool approved by FDA, has been made available for use clinically to aid early detection and to differentiate malignant from benign nodules in lung cancer [34, 35]. Moreover, a randomized controlled trial investigating the role of the clinical and cost effectiveness of this test for lung cancer screening is now being carried out, of which the early results are very encouraging [10, 36]. In this study, measurement of autoantibodies against the panel of six TAAs (p53, NY-ESO-1, PRDX 6, MMP-7, Hsp70, and Bmi-1) exhibited an AUC of 0.786 with a sensitivity of 50.0% and a specificity of 90.5% in the diagnosis of early stage EJA in the training cohort, and the data were further verified in the validation cohort. Our finding indicates that the immune response to TAAs is an early event of the tumorigenesis and progression of EJA, and that the generated autoantibodies targeting the TAAs have the potential to serve as early molecular signatures for the detection of EJA. Moreover, this autoantibody panel potentially demonstrated a better diagnostic sensitivity for early stage EJA patients than markers CEA and CA19-9, which are major serum tumor markers in gastrointestinal cancers currently used in clinical practice. The positive rates of CEA and CA19-9 in EJA patients were reported to be only 20.3% and 12.9%, respectively, and these markers are elevated most commonly in advanced-stage patients [37]. Furthermore, PPV is very important for a test used in early detection of cancer. In this study, the autoantibody panel in the training and validation cohorts exhibits PPVs of 81.7% and 84.3%, respectively, for all EJA patients (35.8% and 63.6% for early stage EJA patients, respectively). As is known to all, the PPV is not intrinsic to a diagnostic test, which depends also on the disease prevalence. Even for very accurate tests, when the prevalence of disease is very low, the PPV is still not high. Globally, EJA is estimated with an incidence rate of approximately 3.3 per 100,000 [38]. Such low prevalence of EJA must lead to a very low PPV. When we set the prevalence standardized to 50% [39], which would allow researchers to avoid the large effect of prevalence on PPV when comparing one diagnostic test with another, the PPVs of the autoantibody panel for early stage EJA would be much better (84.0% and 84.9% in the training cohort and the validation cohort, respectively). Thus, such a serum autoantibody test might be helpful for identifying the cases at high-risk and then targeting the endoscopic examination. On the other hand, the sensitivity of this autoantibody panel seems not high enough to be a screening tool for EJA in general or high-risk populations. For screening purpose, the sensitivity of serum biomarker should be higher to reduce false negative rate. Therefore, we should further identify useful autoantibodies to enhance the sensitivity of our present combined autoantibody assay in the future study.

The diagnostic efficiency for early stage EJA was in accordance with our previous studies on assessing the same autoantibody panel for early stage ESCC and nasopharyngeal carcinoma [13, 14]. This result suggests that if asymptomatic population are detected with positive result of this autoantibody panel, they should be considered at higher risk for suffering from EJA or other cancers like ESCC. Furthermore, we observed that a restricted panel consisting of autoantibodies against p53, NY-ESO-1 and Bmi-1 could obtain similar diagnostic performance for early stage EJA (Fig. 3; Table 2). Our previous studies demonstrated that different restricted combinations in early stage ESCC and nasopharyngeal carcinoma, autoantibodies against p53, NY-ESO-1, PRDX6 and Hsp70, and autoantibodies against p53, NY-ESO-1, Bmi-1 and Hsp70, respectively, kept high sensitivity and specificity in detecting corresponding tumor samples [13, 14]. Previous reports from Japanese researchers provided convincing data, which show that autoantibodies against p53 and NY-ESO-1 are useful biomarkers in the early diagnosis of ESCC and gastric cancer [40–42]. From the above evidence, we could deduce that autoantibodies against p53 and NY-ESO-1 antigens are indispensable to the autoantibody panel assays for these types of tumors. On the other hand, these data also indicate the heterogeneity of cancer, and reveal that the importance of individual autoantibodies in the panel assay varied. This phenomenon raises a question in the field of autoantibodies and cancer detection: how should we choose the right combination in a certain type of cancer that gives the highest sensitivity and specificity? This could be likely resolved by means of proteomic technologies which enabled large numbers of TAAs to be discovered concomitantly [43].

Although autoantibodies have been suggested as promising diagnostic biomarkers, few have been well assessed to be used to monitor therapeutic response or predict prognosis of cancer [43]. In this study, the decrease in autoantibodies levels (i.e., autoantibodies against p53, NY-ESO-1, PRDX6 and Bmi-1, Fig. 4) or in positive rates of the autoantibody panel was observed in serum after surgery, which indicates that autoantibodies might be surveillance biomarkers to evaluate the therapeutic response of EJA patients. We further analyzed prognostic value of individual autoantibodies and the autoantibody panel in EJA, but we found that autoantibodies in EJA were not related to the prognosis (Supplementary Fig. 2). Hoshino et al. [42] also reported that the difference of 3-year survival rates between the autoantibody-positive group and the autoantibody-negative group was not statistically significant. On the contrary, another study reported by Suzuki et al. [44] suggested that high serum titer of p53 autoantibodies was an independent prognosis factor for esophageal cancer patients. Thus, the results of autoantibodies as indicators of cancer prognosis are mixed [43]. Further large sample evaluation and long-term follow-up would help to clarify this question.

A recent study by Zhou et al. [12] evaluated autoantibody against to a panel of seven TAAs (p53, Koc, P62, C-myc, IMP1, Survivn and p16) in EJA, which were different from the TAAs investigated in our present study in all but just p53. The AUC, sensitivity and specificity in EJA reported in their study were 0.73%, 64% and 87%, respectively, which are similar to those we have demonstrated here. However, no detailed data on pathological stage of the cancers were available and the sample size of study population were smaller than those used in this study.

In summary, our data demonstrate that the autoantibody panel or the restricted autoantibody panel could help identify early stage EJA. Although current study results evaluating autoantibody biomarkers for EJA are promising, there are two major limitations in the present study: the small study subjects of early stage EJA and the lack of prospective cohort validation. In the future work, larger number of early stage and prediagnostic EJA samples are needed to validate the ability of these autoantibody assays in early detection of EJA.

## Electronic supplementary material

Below is the link to the electronic supplementary material.

Supplementary material 1 (PDF 770 KB)
