# Supplementary material for: Combined detection of serum autoantibodies as diagnostic biomarkers in esophagogastric junction adenocarcinoma
Source: Gastric Cancer. 2018 Nov 13;22(3):546–57. doi: 10.1007/s10120-018-0894-y (PMC6476828; doi:10.1007/s10120-018-0894-y)
Supplement: Supplementary file 1 — Supplementary material 1 (PDF 770 KB) [file 10120_2018_894_MOESM1_ESM.pdf]

Supplementary Table 1. Diagnostic results for individual autoantibodies in EJA in the training cohort

	AUC (95%CI)	Sensitivity	Specificity	PPV	NPV	PLR	NLR
**EJA vs. NC**							
p53	0.718 (0.659-0.777)	35.2%	90.5%	72.8%	65.9%	3.71	0.72
NY-ESO-1	0.718 (0.658-0.779)	37.7%	90.5%	74.1%	66.8%	3.97	0.69
PRDX6	0.573 (0.502-0.644)	34.4%	90.5%	72.3%	65.7%	3.62	0.72
MMP-7	0.597 (0.527-0.667)	30.3%	90.5%	69.7%	64.3%	3.19	0.77
Hsp70	0.652 (0.589-0.715)	18.0%	90.5%	57.7%	60.5%	1.89	0.91
Bmi-1	0.686 (0.623-0.749)	22.1%	90.5%	62.7%	61.7%	2.33	0.86
**Early-stage EJA vs. NC**							
p53	0.648 (0.498-0.798)	33.3%	90.5%	27.1%	92.7%	3.51	0.74
NY-ESO-1	0.654 (0.507-0.802)	27.8%	90.5%	23.7%	92.2%	2.93	0.80
PRDX6	0.602 (0.436-0.768)	38.9%	90.5%	30.3%	93.3%	4.09	0.68
MMP-7	0.575 (0.406-0.744)	33.3%	90.5%	27.2%	92.7%	3.51	0.74
Hsp70	0.697 (0.581-0.814)	16.7%	90.5%	15.7%	91.1%	1.76	0.92
Bmi-1	0.685 (0.544-0.826)	27.8%	90.5%	23.7%	92.2%	2.93	0.80

CI, exact confidence interval; EJA, esophagogastric junction adenocarcinoma; NC, normal controls; NLR, negative likelihood ratio; NPV, negative predictive value; PLR, positive likelihood ratio; PPV, positive predictive value.

Supplementary Table 2. Diagnostic results for individual autoantibodies in EJA in the validation cohort

	AUC (95%CI)	Sensitivity	Specificity	PPV	NPV	PLR	NLR
**EJA vs. NC**							
p53	0.766 (0.691-0.841)	35.7%	96.3%	72.8%	65.9%	3.71	0.72
NY-ESO-1	0.747 (0.670-0.824)	34.3%	95.0%	74.1%	66.8%	3.97	0.69
PRDX6	0.647 (0.557-0.737)	30.0%	90.0%	72.3%	65.7%	3.62	0.72
MMP-7	0.599 (0.505-0.694)	24.3%	95.0%	69.7%	64.3%	3.19	0.77
Hsp70	0.686 (0.601-0.770)	28.6%	86.3%	57.7%	60.5%	1.89	0.91
Bmi-1	0.711 (0.619-0.803)	54.3%	90.0%	62.7%	61.7%	2.33	0.86
**Early-stage EJA vs. NC**							
p53	0.799 (0.705-0.893)	40.0%	96.3%	77.2%	83.7%	10.81	0.62
NY-ESO-1	0.714 (0.602-0.826)	28.0%	95.0%	63.6%	80.9%	5.60	0.76
PRDX6	0.629 (0.491-0.767)	28.0%	90.0%	46.7%	80.0%	2.80	0.80
MMP-7	0.609 (0.460-0.757)	28.0%	95.0%	63.6%	80.9%	5.60	0.76
Hsp70	0.702 (0.592-0.811)	32.0%	86.3%	42.2%	80.3%	2.34	0.79
Bmi-1	0.682 (0.536-0.829)	40.0%	90.0%	55.5%	82.8%	4.00	0.67

CI, exact confidence interval; EJA, esophagogastric junction adenocarcinoma; NC, normal controls; NLR, negative likelihood ratio; NPV, negative predictive value; PLR, positive likelihood ratio; PPV, positive predictive value.

Supplementary Table S3. Correlation between the autoantibody panel and panel of 3 and clinicopathologic characteristics of EJA patients in the training cohort

	n	the autoantibody panel			panel of 3		
		Positive (%)	*X*	*P*	Positive (%)	*X*	*P*
Patient age							
≤ 60	46	28 (60.9)	1.232	0.267	25 (54.3)	2.608	0.106
> 60	76	44 (57.9)			40 (52.6)		
Patient gender							
Male	103	61 (59.2)	0.096	0.757	56 (54.4)	0.002	0.962
Female	19	11 (57.9)			9 (47.4)		
Size of tumor							
≤ 5 cm	49	31 (63.3)	0.476	0.490	28 (57.1)	0.043	0.836
> 5 cm	65	37 (56.9)			34 (52.3)		
Histological grade							
Grade 1+2	58	33 (56.9)	1.039	0.595	29 (50.0)	1.472	0.479
Grade 3	47	30 (63.8)			28 (59.6)		
Depth of tumor invasion							
T1+T2+T3	34	20 (58.8)	0.791	0.374	18 (52.9)	1.926	0.165
T4	88	52 (59.1)			47 (53.4)		
Lymph node metastasis							
Positive	94	57 (60.6)	0.065	0.799	51 (54.3)	0.804	0.370
Negative	28	15 (53.6)			14 (50.0)		
TNM stage							
Ⅰ+Ⅱ	18	7 (38.9)	0.724	0.696	10 (55.6)	3.347	0.188
Ⅲ+IV	104	63 (60.6)			55 (52.9)		

Supplementary Table S4. Correlation between the autoantibody panel and panel of 3 and clinicopathologic characteristics of EJA patients in the validation cohort

	n	the autoantibody panel			panel of 3		
		Positive (%)	*X*	*P*	Positive (%)	*X*	*P*
Patient age							
≤ 60	24	15 (62.5)	0.018	0.894	14 (58.3)	0.042	0.837
> 60	46	28 (60.9)			28 (60.9)		
Patient gender							
Male	55	34 (61.8)	0.016	0.898	33 (60.0)	0.000	1.000
Female	15	9 (60.0)			9 (60.0)		
Size of tumor							
≤ 5 cm	37	24 (64.9)	0.952	0.329	24 (64.9)	1.694	0.282
> 5 cm	23	12 (52.2)			11 (47.8)		
Histological grade							
Grade 1+2	33	18 (54.5)	2.498	0.114	18 (54.5)	1.555	0.212
Grade 3	24	18 (75.0)			17 (70.8)		
Depth of tumor invasion							
T1+T2+T3	55	31 (56.4)	2.779	0.096	30 (54.5)	3.182	0.074
T4	15	12 (80.0)			12 (80.0)		
Lymph node metastasis							
Positive	45	28 (62.2)	0.033	0.855	28 (62.2)	0.259	0.611
Negative	25	15 (60.0)			14 (56.0)		
TNM stage							
Ⅰ+Ⅱ	24	13 (54.2)	0.813	0.367	13 (54.2)	0.518	0.472
Ⅲ+Ⅳ	46	30 (65.2)			29 (63.0)		

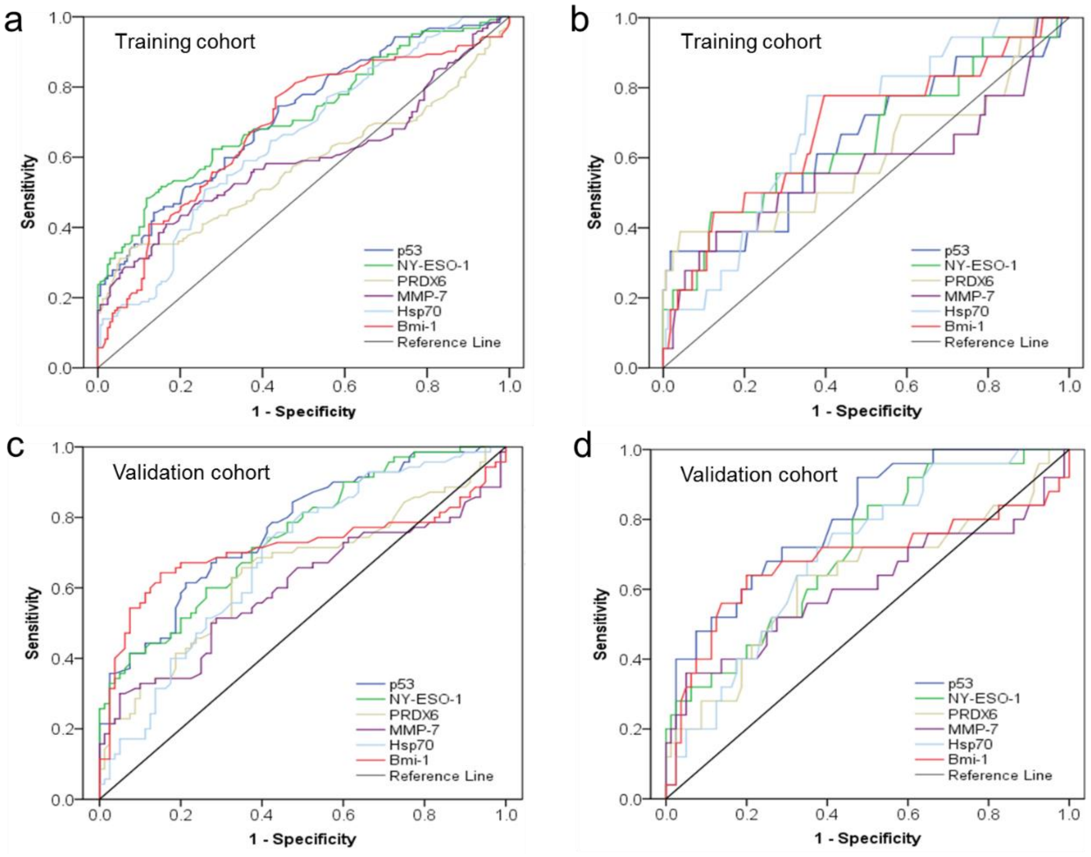

Supplementary Figure 1 Performance of individual autoantibodies to detect EJA. A. ROC curve for autoantibodies against p53, NY-ESO-1, PRDX6, MMP-7, Hsp70 and Bmi-1 for all patients with EJA versus normal controls in training cohort. B. ROC curve for autoantibodies against p53, NY-ESO-1, PRDX6, MMP-7, Hsp70 and Bmi-1 for patients with early-stage EJA versus normal controls in training cohort. C. ROC curve for autoantibodies against p53, NY-ESO-1, PRDX6, MMP-7, Hsp70 and Bmi-1 for all patients with EJA versus normal controls in validation cohort. D. ROC curve for autoantibodies against p53, NY-ESO-1, PRDX6, MMP-7, Hsp70 and Bmi-1 for patients with early-stage EJA versus normal controls in validation cohort. EJA, esophagogastric junction adenocarcinoma.

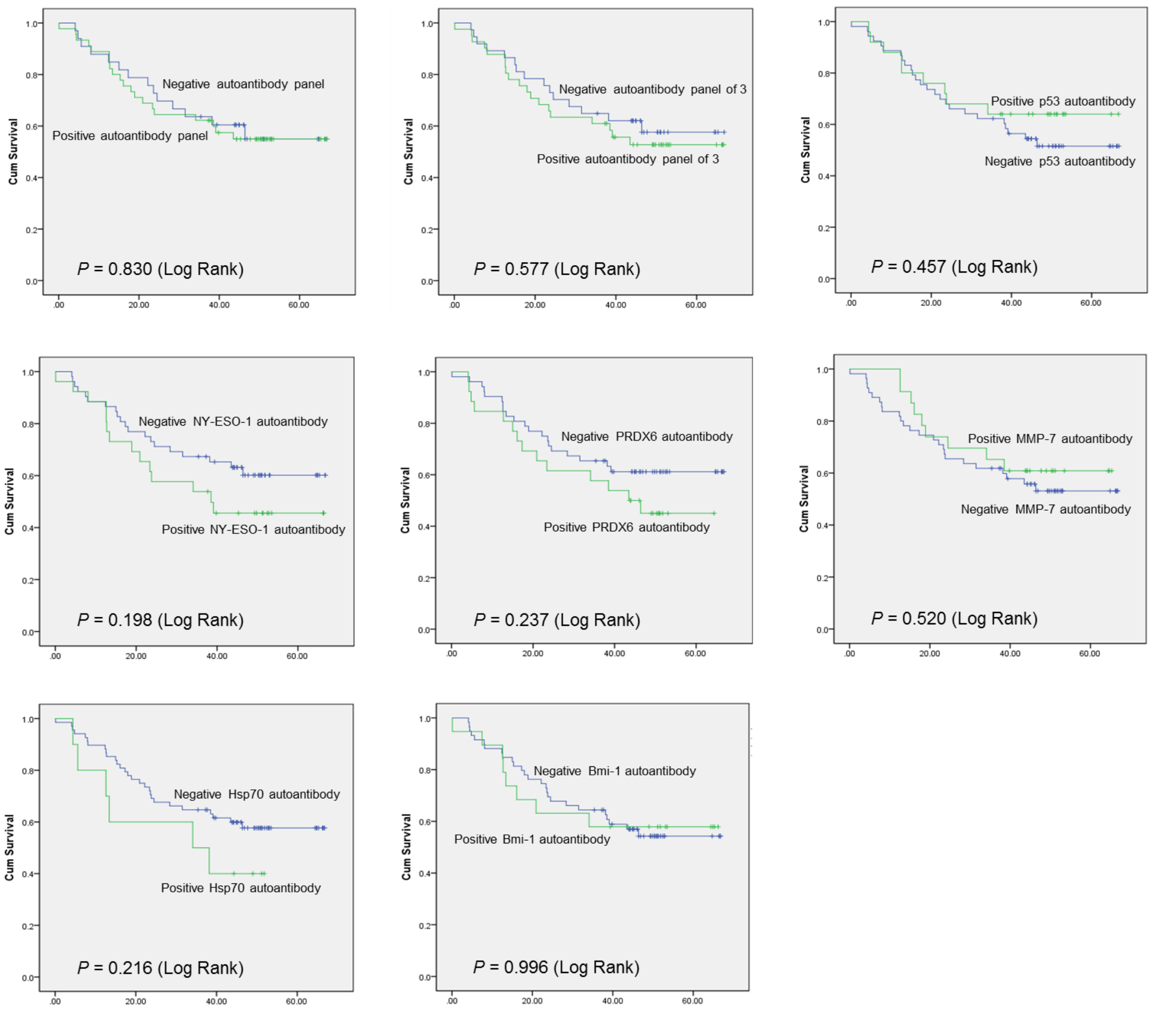

Supplementary Figure 2 Kaplan-Meier analysis of the overall survival of patients with esophagogastric junction adenocarcinoma according to autoantibody levels. Panel: autoantibodies against six tumor-associated antigens. Panel of 3: autoantibodies against p53, NY-ESO-1 and Bmi-1.
